# Supplementary material for: Resolving the full spectrum of human genome variation using Linked-Reads
Source: Genome Res. 2019 Apr;29(4):635–45. doi: 10.1101/gr.234443.118 (PMC6442396; doi:10.1101/gr.234443.118)
Supplement: Supplemental Material [file supp_gr.234443.118_Supplemental_longranger-2.2.zip › longranger-2.2.tar.bz2!/lr2.2-public/lib/go/src/code.google.com/vitess/docs/user-guide/sharding-kubernetes-workflow.html]

Vitess / Sharding in Kubernetes (Tutorial, automated)


Toggle navigation

- Overview
- Guides
- Reference
- Blog
- GitHub

- #### Overview

  - What is Vitess
  - Scaling MySQL with Vitess
  - Key Concepts
- #### Getting Started

  - Run Vitess on Kubernetes
    - Custom Docker Build
  - Run Vitess Locally
- #### User Guide

  - Introduction- Client Libraries- Backing Up Data- Reparenting
        - Schema Management
        - VSchema Guide
        - Schema Swap (Tutorial)
        - Sharding
          - Horizontal Sharding (Tutorial, automated)
          - Horizontal Sharding (Tutorial, manual)
          - Sharding in Kubernetes (Tutorial, automated)
          - Sharding in Kubernetes (Tutorial, manual)
        - Vitess Sequences
        - MySQL Server Protocol
        - Vitess and Replication
        - Update Stream
        - Row Based Replication
        - Topology Service
        - Transport Security Model
        - Launching
          - Scalability Philosophy
          - Production Planning
          - Server Configuration
          - 2PC Guide
          - Troubleshooting
        - Upgrading
- #### Reference Guides

  - Vitess API- vtctl Commands
- #### Other Resources

  - Presentations- Blog- Roadmap
- #### Contributing

  - Contributing to Vitess- GitHub Workflow- Code Reviews
- GitHub

# Sharding in Kubernetes (Tutorial, automated)

- #### Overview

  - What is Vitess
  - Scaling MySQL with Vitess
  - Key Concepts
- #### Getting Started

  - Run Vitess on Kubernetes
    - Custom Docker Build
  - Run Vitess Locally
- #### User Guide

  - Introduction- Client Libraries- Backing Up Data- Reparenting
        - Schema Management
        - VSchema Guide
        - Schema Swap (Tutorial)
        - Sharding
          - Horizontal Sharding (Tutorial, automated)
          - Horizontal Sharding (Tutorial, manual)
          - Sharding in Kubernetes (Tutorial, automated)
          - Sharding in Kubernetes (Tutorial, manual)
        - Vitess Sequences
        - MySQL Server Protocol
        - Vitess and Replication
        - Update Stream
        - Row Based Replication
        - Topology Service
        - Transport Security Model
        - Launching
          - Scalability Philosophy
          - Production Planning
          - Server Configuration
          - 2PC Guide
          - Troubleshooting
        - Upgrading
- #### Reference Guides

  - Vitess API- vtctl Commands
- #### Other Resources

  - Presentations- Blog- Roadmap
- #### Contributing

  - Contributing to Vitess- GitHub Workflow- Code Reviews

This guide shows you an example about how to apply range-based sharding
process in an existing unsharded Vitess keyspace
in Kubernetes using the horizontal resharding workflow.
In this example, we will reshard from 1 shard "0" into 2 shards "-80" and "80-".
We will follow a process similar to the general
Horizontal Sharding guide
except that here we'll give you the commands you'll need in the kubernetes
environment.

## Overview

The horizontal resharding process overview can be found
here

## Prerequisites

You should complete the Getting Started on Kubernetes
guide (please finish all the steps before Try Vitess resharding) and have left
the cluster running. Then, please follow these steps before running the
resharding process:

1. Configure sharding information. By running the command below, we tell
   Vitess to shard the data using the page column through the provided VSchema.

   ```
   vitess/examples/kubernetes$ ./kvtctl.sh ApplyVSchema -vschema "$(cat vschema.json)" test_keyspace
   ```
2. Bring up tablets for 2 additional shards: *test\_keyspace/-80* and
   *test\_keyspace/80-* (you can learn more about sharding key range
   here):

   ```
   vitess/examples/kubernetes$ ./sharded-vttablet-up.sh
   ```

   Initialize replication by electing the first master for each of the new shards:

   ```
   vitess/examples/kubernetes$ ./kvtctl.sh InitShardMaster -force test_keyspace/-80 test-200
   vitess/examples/kubernetes$ ./kvtctl.sh InitShardMaster -force test_keyspace/80- test-300
   ```

   After this set up, you should see the shards on Dashboard page of vtctld UI
   (http://localhost:8001/api/v1/proxy/namespaces/default/services/vtctld:web).
   There should be 1 serving shard named "0" and 2 non-serving shards named
   "-80" and "80-". Click the shard node, you can inspect all its tablets
   information.
3. Bring up a *vtworker* process (a pod in kubernetes) and a *vtworker* service
   which is used by the workflow to connect with the *vtworker* pod. (The
   number of *vtworker* should be the same of original shards, we start one
   vtworker process here since we have only one original shard in this example.)

   ```
   vitess/examples/kubernetes$ ./vtworker-up.sh
   ```

   You can check out the external IP for the vtworker service (please take note
   of this external IP, it will be used for the vtworker address in creating
   the resharding workflow):

   ```
   $ kubectl get service vtworker
   ```

   You can verify this *vtworker* process set up through http://:15032/Debugging.
   It should be pinged successfully. After you ping the vtworker, please click
   "Reset Job". Otherwise, the vtworker is not ready for executing other tasks.

## Horizontal Resharding Workflow

### Create the Workflow

Using the web vtctld UI to create the workflow is the same with steps in local
environment
except for filling the "vtworker Addresses" slot. You need to get the external
IP for vtworker service (mentioned in
Prerequisites) and use
<EXTERNAL-IP>:15033 as the vtworker addresses.

Another way to start the workflow is through the vtctlclient command:

```
vitess/examples/kubernetes$ ./kvtctl.sh WorkflowCreate -skip_start=false horizontal_resharding -keyspace=test_keyspace -vtworkers=<EXTERNAL-IP>:15033 -enable_approvals=true
```

### Approvals of Tasks Execution (Canary feature)

Please check the content in general
Horizontal Sharding guide

### Retry

Please check the content in general
Horizontal Sharding guide

### Checkpoint and Recovery

Please check the content in general
Horizontal Sharding guide

## Verify Results and Clean up

After the resharding process, data in the original shard is identically copied
to new shards. Data updates will be visible on the new shards, but not on the
original shard. You should then see in the vtctld UI *Dashboard* page that shard
*0* becomes non-serving and shard *-80* and shard *80-* are serving shards.
Verify this for yourself: inspect the database content,
then add messages to the guestbook page and inspect again. You can use
http://<EXTERNAL-IP> (EXTERNAL-IP refers to the external IP of the guest book
service) to visit the guestbook webpage in your browser and choose any random
page for inserting information. Details can be found
here)
You can inspect the database content using the following commands:

```
# See what's on shard test_keyspace/0
# (no updates visible since we migrated away from it):
vitess/examples/kubernetes$ ./kvtctl.sh ExecuteFetchAsDba test-100 "SELECT * FROM messages"
# See what's on shard test_keyspace/-80:
vitess/examples/kubernetes$ ./kvtctl.sh ExecuteFetchAsDba test-200 "SELECT * FROM messages"
# See what's on shard test_keyspace/80-:
vitess/examples/kubernetes$ ./kvtctl.sh ExecuteFetchAsDba test-300 "SELECT * FROM messages"
```

You can also checkout the *Topology* browser on vtctl UI. It shows you the
information of the keyrange of shard and their serving status. Each shard
should look like this

shard 0

shard -80

shard 80-

After you verify the result, we can remove the
original shard since all traffic is being served from the new shards:

```
vitess/examples/kubernetes$ ./vttablet-down.sh
```

Then we can delete the now-empty shard:

```
vitess/examples/local$ ./kvtctl.sh DeleteShard -recursive test_keyspace/0
```

You should then see in the vtctld UI *Dashboard* page that shard *0* is gone.

## Tear down and Clean up

Since you already cleaned up the tablets from the original unsharded example by
running `./vttablet-down.sh`, that step has been replaced with
`./sharded-vttablet-down.sh` to clean up the new sharded tablets.

```
vitess/examples/kubernetes$ ./guestbook-down.sh
vitess/examples/kubernetes$ ./vtworker-down.sh
vitess/examples/kubernetes$ ./vtgate-down.sh
vitess/examples/kubernetes$ ./sharded-vttablet-down.sh
vitess/examples/kubernetes$ ./vtctld-down.sh
vitess/examples/kubernetes$ ./etcd-down.sh
```

Then tear down the Container Engine cluster itself, which will stop the virtual machines running on Compute Engine:

```
$ gcloud container clusters delete example
```

It's also a good idea to remove the firewall rules you created, unless you plan to use them again soon:

```
$ gcloud compute firewall-rules delete vtctld guestbook
```

## Reference

You can checkout the old version tutorial here.
It walks you through the resharding process by manually executing commands.

For the kubectl command line interface, which helps you interact with the
kubernetes cluster, you can check out more information
here.

## Troubleshooting

### Checking status of your setup.

```
To get status of pods and services you've setup, you can use the commands
(all pods should be in Running status, guestbook and vtworker services
should have assign external IP):

``` sh
$ kubectl get pods
$ kubectl get services
```
```

### Debugging pods.

```
If you find out a component (e.g. vttablet, vtgate) doesn't respond as
expected, you can surface the log using this command (the pod name can be
found out using the command mentioned above):

``` sh
$ kubectl logs <pod name> [-c <container>]
### example
# $ kubectl logs vtworker
# $ kubectl logs vttablet-XXXX -c vttablet
```
```

### Debugging pending external IP issue.

```
If you found that your service has a pending external IP for long time, it
maybe because you've reached the limitation of networking resource. Please
go to your project console on gcloud (cloud.google.com), then go to *Load
balancing* page (you can search "Load balancing" in the search bar to get
to the page) under Networking section. Then, click "advanced menu" for
editing load balancing resources. Check the forwarding rules you have and
delete the unused ones if there are too many.
```

Contact: vitess@googlegroups.com  **·**  
Announcements  **·**  
© 2017 Vitess powered by Google Inc
